# Priorities of people living with Alzheimer's and care partners: What Matters Most?

**DOI:** 10.1002/alz.71554

**Published:** 2026-08-27

**Authors:** Carla (DeMuro) Romano, Emily Bratlee‐Whitaker, Ann Hartry, Jim Taylor, Leigh F. Callahan, Doreen Monks, Ian Kremer, Debra Lappin, Terry Frangiosa, Sanjyot Sangodkar, Jae Lee, Elaheh Shirneshan, Diana Slowiejko, Dana DiBenedetti, William L. Herring, Cooper Bussberg, Gabrielle J. Dardis, Diana Goss, Teresa Edwards, Lori McLeod, Christine Poulos, Russ Paulsen

**Affiliations:** ^1^ RTI Health Solutions Durham North Carolina USA; ^2^ Eli Lilly & Co Indianapolis Indiana USA; ^3^ Voices of Alzheimer's (VoA) Washington DC USA; ^4^ Department of Medicine University of North Carolina Chapel Hill North Carolina USA; ^5^ Advocate and person living with Alzheimer's disease Livingston New Jersey USA; ^6^ LEAD Coalition (Leaders Engaged on Alzheimer's Disease) Washington District of Columbia USA; ^7^ Lappin Kramer LLC Denver Colorado USA; ^8^ Faegre Drinker Consulting Washington District of Columbia USA; ^9^ AbbVie Inc. Chicago Illinois USA; ^10^ Genentech San Francisco California USA; ^11^ Karolinska Institute Stockholm Sweden; ^12^ UsAgainstAlzheimer's Washington District of Columbia USA

**Keywords:** Alzheimer's disease, care partner, outcomes, patient‐centered, patient experience, treatment benefit, What Matters Most

## Abstract

**INTRODUCTION:**

Understanding the experience of people living with Alzheimer's disease (PLWAD) and care partners is central to defining meaningful treatment outcomes. The What Matters Most (WMM) research program seeks to identify and measure treatment‐related needs, preferences, and priorities across the disease continuum.

**METHODS:**

This mixed‐methods, observational, US‐based study included qualitative interviews and a cross‐sectional, web‐based quantitative survey assessing priorities among WMM model concepts and domains.

**RESULTS:**

Racially and ethnically diverse participants represented the full spectrum of disease severity. Qualitative interviews (*N* = 64) supported a WMM conceptual model of disease comprising 50 concepts across six domains: General Independence, Thought Processing, Communication, Daily Activities, Emotions, and Social Life/Activities. All WMM concepts were deemed important, but the quantitative survey priority ranking (*N* = 640) identified differences in prioritization among PLWAD and care partners.

**DISCUSSION:**

These findings provide novel, critical insights into the lived experience of Alzheimer's disease (AD) and the identification of meaningful treatment outcomes.

## Highlights

- WMM research includes PLWAD and care partners across the AD severity continuum.
- A representative and diverse population of US PLWAD and care partners was recruited.
- The refined conceptual model of disease comprised 50 WMM concepts across six domains.
- PLWAD and care partners endorsed the importance of all WMM concepts.
- Concept and domain priorities differed meaningfully between PLWAD and care partners.

## 1 INTRODUCTION

Alzheimer's disease (AD) is a devastating, progressive, and ultimately fatal disease affecting not only the people at risk or with a diagnosis but also their care partners, family members and loved ones, and society as a whole.^1^ Emerging therapies for AD aim to demonstrate meaningful benefits as measured by neuropsychological evaluations and other clinical outcome assessments (COAs) in clinical trials and observational study settings. COAs may employ continuous evaluations or be event‐based to capture change in disease progression. However, while designed to capture subtle change in cognition, existing assessments may not consider or fully capture many of the outcomes or concepts inherently meaningful to people living with AD (PLWAD), those at high risk of developing AD, and care partners – including emotional and psychological impacts associated with AD – or the differences in treatment‐related priorities for individuals across the continuum of AD severity.^2^, ^3^, ^4^, ^5^, ^6^ A greater understanding of the experiences, needs, and priorities of PLWAD and care partners across the continuum of the disease is central to defining clinically meaningful treatment outcomes. This understanding informs the development of AD treatments and programs, as well as regulatory reviews, health technology assessments, and reimbursement decisions for new drugs and services to treat and manage AD. Seeking this understanding is consistent with patient‐focused drug development in the United States (US)^7^, ^8^, ^9^, ^10^ and use of patient experience data globally.

The What Matters Most (WMM) research program, sponsored by the AD Patient and Caregiver Engagement (AD PACE) initiative of UsAgainstAlzheimer's, is a series of studies seeking to characterize the lived experience of PLWAD and care partners and to identify and measure treatment‐related needs, preferences, and priorities across the full spectrum of AD and among a diverse and inclusive US participant population. Previous mixed‐methods WMM research identified core signs, symptoms, and behaviors of the lived experience that were elicited from PLWAD and care partners (the original 42 WMM concepts^11^). The importance of those concepts was quantified with individuals across the disease severity spectrum,^12^ and a draft conceptual model of disease categorizing the original WMM concepts within hypothesized domains was developed.^13^ Further, the original WMM concepts were mapped against 20 COAs most frequently used in AD, and measurement gaps were noted between the concepts assessed by these COAs and WMM concepts.^14^

The current WMM research reported in this article builds upon those previous studies by first refining the original 42 concepts and the conceptual model informed by qualitative interviews, followed by quantitatively prioritizing the concepts and their domains among a large (*N* ≥ 600) racially and ethnically diverse cohort of PLWAD and care partners across the full AD severity spectrum. The quantitative component also included measurement of health‐related quality of life (HRQOL), assessment of the impacts of WMM concepts on daily life, and an initial evaluation of psychometric properties of the hypothesized conceptual model domains to inform a roadmap from WMM concepts to COA. The reporting and analysis of those aspects of the work are beyond the scope of this article; the results reported herein describe the qualitative refinement of the conceptual model of disease and the quantitative evaluation of ranked priorities among WMM concepts and conceptual model domains by PLWAD and care partners across the AD severity spectrum.

**RESEARCH IN CONTEXT**

1. **Systematic review**: This observational study in the WMM research program included qualitative interviews evaluating a conceptual model of disease and a quantitative survey assessing priorities among model domains and concepts with PLWAD and care partners representing the full disease severity continuum.
2. **Interpretation**: Similarities and differences in concept and domain prioritization between PLWAD and care partners and by disease severity were observed. Simultaneously and consistent with previous research, PLWAD and care partners endorsed all WMM concepts as important, which supports the relevancy of WMM concepts while demonstrating the impact of lived experience on prioritization.
3. **Future directions**: Insights from this novel dataset can inform critical future research directions, including explorations of clinical meaningfulness and priority shifts over the course of disease progression, ultimately informing the selection or development of outcome measures and endpoints assessing the meaningful benefits of AD treatments.

## 2 METHODS

### 2.1 Study design

This US‐based mixed‐methods, observational study was informed by previous WMM work^11^, ^12^, ^14^ and included several stages. Stage 1 involved conducting a new series of qualitative interviews to inform refinement of a conceptual model of disease. Stage 2a involved the development, pretesting, and cognitive debriefing of a draft, cross‐sectional, web‐based quantitative survey, and Stage 2b involved administration of the final quantitative survey to a target sample of ≥600 adults aged ≥18 years representing diverse racial and ethnic identities living with or at risk of AD and care partners of individuals across the full spectrum of disease. All applicable study materials were distributed in English or US Spanish. This study was reviewed by the RTI International Institutional Review Board (IRB) and was determined to meet the criteria for exemption from IRB review.

The quantitative survey (Stage 2) included multiple components to address each study aim; components assessing HRQOL, the impact of concepts, and psychometric properties are not reported in this manuscript. In alignment with good research practices and the methodological literature, the WMM concept and domain prioritization component used best‐worst scaling (BWS), a method increasingly applied to prioritize patient‐centered outcomes to support clinical trial design.^15^, ^16^, ^17^, ^18^, ^19^, ^20^ Object‐case BWS presented survey respondents with a series of choice questions, each with a list of the WMM domains or a subset of the WMM concepts, and asked respondents to select the most important and least important concepts from the subset listed. The concepts in each question and the total number of questions were determined by a balanced, incomplete block experimental design (BIBD) for each WMM domain to ensure that within each domain, each concept and each pair of concepts were presented an equal number of times. The BIBD ensures that the amount of prioritization information collected about each concept is consistent, which helps to ensure the validity of prioritization data. For some domains that required more BWS questions to elicit priorities, participants were randomly assigned to a subset of the questions to minimize participant burden.

### 2.2 Study population and recruitment

Understanding that signs, symptoms, behaviors, impacts, treatment‐related needs, preferences, and priorities in AD may change over the course of the disease, and to ensure that the full range of these constructs was assessed, all data collection activities across stages included participants belonging to each of five WMM AD groups as described in Table S1, including approximate mapping to US Food and Drug Administration (FDA)‐defined AD stages from Early AD: Developing Drugs for Treatment draft guidance.^21^ These include clinically diagnosed PLWAD in Groups 1 to 3 (Group 1, individuals with non‐clinically impaired cognition who are at high risk of developing AD or who have evidence of AD pathology; Group 2, individuals diagnosed with mild cognitive impairment [MCI] due to AD; Group 3, individuals diagnosed with mild AD dementia) and care partners in Groups 4 and 5 (Group 4, care partners of individuals diagnosed with moderate AD dementia; Group 5, care partners of individuals diagnosed with severe AD dementia).

Importantly, clinicians verified participant group classification in order to include participants at risk of or with MCI or with dementia due to AD specifically (Table S1). For Group 1, clinicians verified AD pathology as determined by genetic risk or positive findings of an amyloid positron emission tomography scan or cerebrospinal fluid lumbar puncture within the past 6 months; for Group 2, a diagnosis of MCI due to AD was determined by the same evidence of AD pathology accompanied by self‐report or chart notes describing memory problems, losing or misplacing things, and so forth; for Groups 3 to 5, diagnoses of mild, moderate, or severe AD were determined according to Mini‐Mental State Examination (MMSE) scores, a physician's assessment, or a comparable neuropsychological assessment within the past 6 months.

Across study stages, participants were required to be able to read, write, and understand English or Spanish and, as applicable, be able to participate in 60‐min interviews (full eligibility criteria are reported in Table S2). Participants were excluded if there was a history of other types of dementia (e.g., dementia due to Parkinson's disease, Huntington's disease, Lewy body dementia), a recent history (past year) of traumatic brain injury that was considered the primary cause for dementia, or the presence of any mental or other medical condition that the participant's physician felt would interfere with a PLWAD participant's ability to engage in an interview or survey. Originally, vascular dementia was considered an exclusion criterion. However, in the course of quantitative survey administration (Stage 2b), recruitment partners reported after approximately 350 participants were recruited that Black and Hispanic participants were disproportionately excluded due to a comorbid diagnosis of vascular dementia. Following a review of the literature and gathering of expert consensus – including seeking a non‐binding opinion from the US FDA representatives in a virtual meeting on November 22, 2024 – exclusion criteria were revised to allow inclusion of participants with comorbid vascular dementia.

Recruitment partners (Table S3) targeted representativeness in line with the US population across races and ethnicities, sexes, and educational levels both across the sample and within each of the five AD groups. Clinical confirmation was determined by clinical diagnoses of AD (or defined as “at risk” based on genetic risk or evidence of AD pathology for Group 1). Participants were recruited by specialty qualitative research organizations, clinical trial sites, and a patient advocacy organization (UsAgainstAlzheimer's A‐LIST). The quantitative survey sample was specifically targeted to include ≥50% of participants overall and within each of the five AD groups who self‐identified as African American or Black; Alaska Native; American Indian or Native American; Asian or Asian American; Hispanic; Latina/o, Latine, or Latinx; Middle Eastern and/or North African; Native Hawaiian or Pacific Islander; or a multiracial identity. Recruitment specifically targeted oversampling of Black and Hispanic participants to facilitate comparisons of results across participant subgroups.

### 2.3 Qualitative interviews (Stage 1)

In‐depth, individual, web‐based qualitative interviews evaluated the original 42 WMM concepts to confirm and supplement previous WMM findings^11^, ^12^ and to inform the development of the subsequent quantitative survey (Stage 2). Interviews were conducted by two experienced qualitative research staff between February 6, 2023, and March 30, 2023, using standardized, semi‐structured interview guides for PLWAD or care partners to ensure consistency and comparability and designed to be approximately 60 min in length, with breaks offered as needed to participants. All interviews were audio recorded, then transcribed verbatim and deidentified for analysis. Oral informed consent (and assent for individuals who could not provide consent due to cognitive impairments) was obtained from all participants prior to the start of the interview. A readiness checklist was used to evaluate participant disposition, engagement, and willingness to participate for both PLWAD and care providers. Each interview began with a brief concept elicitation encouraging the participant to speak about their experiences with memory or thinking problems. This was followed by targeted questions to evaluate participant interpretation of the original 42 WMM concepts^11^ and the content of the draft conceptual model. Additionally, participants were asked about their thoughts on the question‐and‐answer process to evaluate aspects of WMM concepts other than importance (i.e., bother, interference, impact) and evaluated a ranking exercise among WMM concepts presented in sets of two, three, or six options.

### 2.4 Draft survey and cognitive debriefing (Stage 2a)

Informed by qualitative interviews, a draft web‐based quantitative survey was developed using distinct sections to assess each aim (e.g., BWS to facilitate priority ranking) and then pretested and debriefed in interviews with a convenience sample of PLWAD and care partners to examine survey comprehension and feasibility. Draft survey pretesting and cognitive debriefing interviews were conducted between February 20, 2024, and April 19, 2024. The draft survey was shared via web link with participants for completion within the 48 h preceding their scheduled cognitive debriefing interview. Cognitive debriefing interviews were led by a survey methodologist using standardized, semi‐structured guides and conducted sequentially so that any suggested modifications to the survey from each interview were able to be assessed in subsequent interviews. Each interview began with a broad question asking the participant to share their overall impressions of the survey experience, followed by more direct questions about survey length, appearance, navigation, and ease of use. The interviewer then displayed the survey on the shared screen and collected feedback on format, instructions (including the intended reporting perspective for care partner respondents), individual survey items, and scaling options as appropriate. Select revised survey items were shared in subsequent interviews for an abbreviated cognitive debriefing to assess the respondent question‐and‐answer process, optimize survey wording, formatting, and response choices, and confirm ecological validity. The individual survey items and scaling options and the overall survey language, format, and instructions were refined based on the debriefing results.

### 2.5 Final quantitative survey (Stage 2b)

The web‐based quantitative survey was finalized based on draft survey pretesting and cognitive debriefing results. Two versions of the survey were developed: one for PLWAD with no more than mild AD (Groups 1 to 3) who completed the survey on behalf of themselves and another for care partners (Groups 4 and 5) who responded in consideration of an individual with moderate or severe AD for whom they provide care (e.g., “Specifically, we want to know how important each of these impacts is to care partners of individuals living with AD while thinking about the person for whom you provide care”). Participant data collected in the final survey were deidentified using a predefined unique participant identification number, and survey results were reported only in aggregate. After all survey data were collected, the collection system was locked to prevent any changes, and the data were extracted for analysis.

The survey comprised four sections, including (1) background information about the participant, (2) a BWS ranking exercise of 50 WMM concepts and six domains, (3) assessment of impact of the 50 WMM concepts (not presently reported), and (4) participant completion of COAs included to facilitate HRQOL and health economic assessments as well as psychometric evaluation of the proposed domain structure (not presently reported). Background information collected about participants included race and ethnicity, sex, age, and general questions asking PLWAD and/or care partners to describe their concerns with, or experiences of, cognitive or functional issues related to AD, if any. For prioritization of the WMM concepts, each participant answered 17 BWS questions selected in alignment with the experimental design, with each question asking them to identify the most and least important of a subset of three WMM concepts within a given domain, as well as to identify the most and least important among the domains.

### 2.6 Data analysis

Qualitative interview data were systematically reviewed and analyzed. Deidentified interview transcripts were coded, and qualitative content analysis and thematic analysis methods were applied.^22^, ^23^, ^24^, ^25^ Important concepts and dominant trends were identified in each interview and compared across interviews to enable the assessment of patterns in participants’ responses.^26^ Descriptive statistical analyses were performed as appropriate (e.g., mean, standard deviation, range), including those for the frequency (e.g., number, percentages) of select items from the qualitative data.

For the quantitative survey, descriptive statistical analyses were performed for demographics, medical history, and current symptoms for (1) the overall survey sample, (2) each AD group separately, and (3) all PLWAD (Groups 1 to 3) and all care partners (Groups 4 and 5). For continuous and ordinal variables, the number of available observations, mean, standard deviation, median, minimum, and maximum were reported. For categorical variables, the number of available observations and frequency and percentage in each response category were reported. Statistical tests used a significance level of 0.05.

BWS prioritization responses were summarized descriptively, and relative priority weights were summarized for the overall sample, for the PLWAD and care partner cohorts, and for the AD severity group and race and ethnicity subgroups. Race and ethnicity subgroups were defined by the self‐reported identities of PLWAD in Groups 1 to 3 and of care partners in Groups 4 and 5 and by the proxy‐reported identities of PLWAD by care partners in Groups 4 and 5. Relative priority weights for concepts (e.g., how much more important participants find one concept compared with another) were calculated using data on the number of times each concept was selected as “best” (*B*), or most important (i.e., “Select the concept that is most important to you [or the person you provide care for] to either retain or be treated for at this time”), and the number of times each concept was selected as “worst” (*W*), or least important (i.e., “Select the concept that is least important to you [or the person you provide care for] to either retain or be treated for at this time”), within the sample or subgroup of interest. Standardized, relative priority weights were calculated.^27^

Following these methods provides standardized, relative priority weights that sum to 100% for each set of WMM concepts or domains presented. Greater weights correspond to higher assigned priority, and the order of weight magnitudes indicates the ranking of concepts or domains, allowing identification of the items of highest and lowest priority within a particular comparison set (e.g., Concept 1 in Domain 1 is ranked higher than Concept 2 in Domain 1 but cannot be compared against Concept 2 in Domain 2). Further, the relative priority weight magnitudes describe the prioritization of each concept item relative to the other items in the domain (e.g., Concept 1 in Domain 1 with a weight of 30 has a priority that is two times greater than that of Concept 2 in Domain 1 with a weight of 15).

## 3 RESULTS

### 3.1 Participant characteristics

#### 3.1.1 Qualitative and cognitive debriefing interviews (Stage 1‐2a)

A total of 64 PLWAD and care partners participated in qualitative interviews (Stage 1; Tables S4 and S5). Interview participants were diverse across races and ethnicities, sexes, ages, and education levels, with more than half of participants (54% of PLWAD and 53% of care partners) identifying as African American or Black, Asian or Asian American, and/or Hispanic, Latin American, Latine, or Latinx. PLWAD were of mean age 62.8 years (range: 47.0 to 81.0 years), and care partners were of mean age 53.4 years (range: 20.0 to 79.0 years); most PLWAD (75.0%) and care partners (70.0%) were female. Most PLWAD (70.8%) reported living with a spouse or partner, and care partners were most often a member of the patient's immediate or extended family (90.0%).

Draft survey pretesting and cognitive debriefing interviews (Stage 2a) included a convenience sample of 17 PLWAD (*n* = 14) and care partner (*n* = 3) participants, including three participants in each AD group except for Group 2, which had five participants. Over half of participants in the convenience sample were male (11/17 [64.7%]). Among the nine participants with available race or ethnicity data, three (33.3%) identified as African American or Black, one (11.1%) identified as Hispanic, and five (55.6%) identified as White.

#### 3.1.2 Quantitative survey (Stage 2b)

A total of 640 participants consented and completed the web‐based survey, including 375 PLWAD who were at risk of or with no more than mild AD dementia (Group 1, *n* = 134; Group 2, *n* = 120; Group 3, *n* = 121) and 265 care partners of individuals with moderate or severe AD dementia (Group 4, *n* = 133; Group 5, *n* = 132). For Groups 4 and 5, care partners reported characteristics for themselves (“What race[s] or ethnicity[ies] do you consider yourself to be?”) and as proxies for the PLWAD with moderate or severe AD dementia (“Thinking of the person for whom you provide care, what race[s] or ethnicity[ies] does this person consider themselves?”). Overall and within each AD group, the respondent pools represented diverse race and ethnicity, sex, age, and educational status groups, similar to those living with AD in the US (Tables 1 and 2).^1^ Notably exceeding anticipated study enrollment targets, more than half of participants (65.6% of PLWAD and 58.1% of care partners) identified as African American or Black; Alaska Native; American Indian or Native American; Asian or Asian American; Hispanic; Latina/o, Latine, or Latinx; Middle Eastern and/or North African; or Native Hawaiian or Pacific Islander.

**TABLE 1:** Quantitative survey: PLWAD characteristics (*N* = 640).

	PLWAD respondents			PLWAD as reported by care partner respondents	
Question	Group 1 (*n* = 134)	Group 2 (*n* = 120)	Group 3 (*n* = 121)	Group 4 (*n* = 133)	Group 5 (*n* = 132)
**Age**					
Mean (SD)	58.5 (13.2)	61.1 (14.0)	65.8 (10.5)	78.0 (8.2)	76.6 (8.8)
Min‐max	18.0 to 87.0	18.0 to 98.0	32.0 to 84.0	52.0 to 99.0	53.0 to 96.0
**Sex assigned at birth**					
Female	79 (59.0)	66 (55.0)	63 (52.1)	76 (57.1)	71 (53.8)
Male	55 (41.0)	53 (44.2)	58 (47.9)	55 (41.4)	59 (44.7)
Intersex	0 (0.0)	1 (0.8)	0 (0.0)	0 (0.0)	1 (0.8)
Prefer not to answer	0 (0.0)	0 (0.0)	0 (0.0)	2 (1.5)	1 (0.8)
**Race or ethnicity (Please select all that apply)**					
African American or Black	41 (30.6)	46 (38.3)	48 (39.7)	46 (34.6)	42 (31.8)
Alaska Native, American Indian, or Native American	4 (3.0)	3 (2.5)	2 (1.7)	4 (3.0)	1 (0.8)
Asian or Asian American	6 (4.5)	7 (5.8)	0 (0.0)	4 (3.0)	8 (6.1)
Hispanic, Latina/o, Latine, or Latinx	32 (23.9)	26 (21.7)	27 (22.3)	22 (16.5)	26 (19.7)
Middle Eastern and/or North African	0 (0.0)	1 (0.8)	2 (1.7)	0 (0.0)	1 (0.8)
Native Hawaiian or Pacific Islander	0 (0.0)	1 (0.8)	0 (0.0)	0 (0.0)	0 (0.0)
White	55 (41.0)	40 (33.3)	48 (39.7)	62 (46.6)	57 (43.2)
A race or ethnicity not listed	1 (0.7)	2 (1.7)	0 (0.0)	0 (0.0)	0 (0.0)
Prefer not to answer	0 (0.0)	0 (0.0)	0 (0.0)	1 (0.8)	0 (0.0)
**Current marital status**					
Single/never married	32 (23.9)	32 (26.7)	25 (20.7)	18 (13.5)	10 (7.6)
Married/living as married/civil partnership	77 (57.5)	63 (52.5)	52 (43.0)	56 (42.1)	55 (41.7)
Divorced or separated	22 (16.4)	19 (15.8)	24 (19.8)	17 (12.8)	17 (12.9)
Widowed/surviving partner	3 (2.2)	6 (5.0)	20 (16.5)	41 (30.8)	49 (37.1)
Other	0 (0.0)	0 (0.0)	0 (0.0)	0 (0.0)	1 (0.8)
Prefer not to answer	0 (0.0)	0 (0.0)	0 (0.0)	1 (0.8)	0 (0.0)
**With whom do you live? (Select one)**					
Alone, in own home or apartment	47 (35.1)	45 (37.5)	51 (42.1)	19 (14.3)	11 (8.3)
With spouse/partner in our own home	74 (55.2)	62 (51.7)	50 (41.3)	36 (27.1)	38 (28.8)
With spouse/partner in their home	0 (0.0)	0 (0.0)	0 (0.0)	14 (10.5)	5 (3.8)
With adult child or children	9 (6.7)	3 (2.5)	11 (9.1)	32 (24.1)	37 (28.0)
With another relative (not spouse/partner or child)	4 (3.0)	6 (5.0)	3 (2.5)	10 (7.5)	10 (7.6)
With a friend or nonprofessional care partner	0 (0.0)	1 (0.8)	4 (3.3)	3 (2.3)	2 (1.5)
In an assisted living facility	0 (0.0)	1 (0.8)	0 (0.0)	12 (9.0)	22 (16.7)
In a nursing home or rehabilitation facility	0 (0.0)	0 (0.0)	0 (0.0)	3 (2.3)	3 (2.3)
In another type of living situation	0 (0.0)	1 (0.8)	2 (1.7)	3 (2.3)	4 (3.0)
Prefer not to answer	0 (0.0)	1 (0.8)	0 (0.0)	1 (0.8)	0 (0.0)
**Highest grade or level of education**					
Less than high school	3 (2.2)	3 (2.5)	5 (4.1)	9 (6.8)	10 (7.6)
High school diploma or equivalent (e.g., GED)	15 (11.2)	12 (10.0)	23 (19.0)	52 (39.1)	48 (36.4)
Associate's degree/technical school	11 (8.2)	10 (8.3)	9 (7.4)	16 (12.0)	18 (13.6)
Some college	30 (22.4)	28 (23.3)	31 (25.6)	17 (12.8)	30 (22.7)
College degree (e.g., BA, BS)	35 (26.1)	37 (30.8)	36 (29.8)	23 (17.3)	13 (9.8)
Some graduate school but no degree	7 (5.2)	3 (2.5)	4 (3.3)	1 (0.8)	4 (3.0)
Graduate or professional degree (e.g., MS, MD, PhD, JD)	33 (24.6)	27 (22.5)	13 (10.7)	14 (10.5)	8 (6.1)
Prefer not to answer	0 (0.0)	0 (0.0)	0 (0.0)	1 (0.8)	1 (0.8)
**Are you concerned that you have a problem with memory or thinking?**					
Yes	109 (81.3)	116 (96.7)	117 (96.7)	–	–
No	19 (14.2)	2 (1.7)	2 (1.7)	–	–
Don't know or not sure	6 (4.5)	2 (1.7)	2 (1.7)	–	–
**[If response to the above question is No or Don't know or Not sure] Have other people told you they have noticed changes in your memory and thinking or changes in your ability to do everyday tasks?**					
*n*	25	4	4	–	–
Yes	5 (20.0)	3 (75.0)	2 (50.0)	–	–
No	20 (80.0)	1 (25.0)	2 (50.0)	–	–
Don't know or not sure	0 (0.0)	0 (0.0)	0 (0.0)	–	–
**Discussed your memory or thinking problems with your doctor or other healthcare provider?**					
Yes	106 (79.1)	109 (90.8)	115 (95.0)	100 (75.2)	103 (78.0)
No	28 (20.9)	11 (9.2)	6 (5.0)	33 (24.8)	29 (22.0)
**Type of physician providing diagnosis of memory or thinking problems**					
Counselor	0 (0.0)	0 (0.0)	0 (0.0)	0 (0.0)	0 (0.0)
Family medicine	0 (0.0)	0 (0.0)	0 (0.0)	0 (0.0)	0 (0.0)
Internist	0 (0.0)	0 (0.0)	0 (0.0)	0 (0.0)	0 (0.0)
Neurologist	31 (23.1)	53 (44.2)	53 (43.8)	86 (64.7)	85 (64.4)
Primary care/general practitioner	83 (61.9)	64 (53.3)	68 (56.2)	42 (31.6)	43 (32.6)
Other	20 (14.9)	3 (2.5)	0 (0.0)	5 (3.8)	4 (3.0)
**Diagnosed by a healthcare provider with any of the following conditions? (Select all that apply)**					
Age‐related macular degeneration	1 (0.7)	0 (0.0)	0 (0.0)	0 (0.0)	0 (0.0)
Asthma	1 (0.7)	0 (0.0)	0 (0.0)	0 (0.0)	0 (0.0)
Anxiety	43 (32.1)	35 (29.2)	23 (19.0)	24 (18.0)	24 (18.3)
Bipolar 2	0 (0.0)	0 (0.0)	0 (0.0)	1 (0.8)	0 (0.0)
Chronic kidney disease	1 (0.7)	0 (0.0)	0 (0.0)	0 (0.0)	0 (0.0)
COPD	3 (2.2)	10 (8.3)	16 (13.2)	5 (3.8)	18 (13.7)
Depression	37 (27.6)	35 (29.2)	28 (23.1)	33 (24.8)	27 (20.6)
Glaucoma	6 (4.5)	6 (5.0)	5 (4.1)	10 (7.5)	13 (9.9)
High blood pressure (hypertension)	43 (32.1)	42 (35.0)	45 (37.2)	65 (48.9)	65 (49.6)
High cholesterol	23 (17.2)	24 (20.0)	23 (19.0)	35 (26.3)	26 (19.8)
Osteoporosis	5 (3.7)	8 (6.7)	12 (9.9)	17 (12.8)	11 (8.4)
Osteoarthritis	12 (9.0)	16 (13.3)	8 (6.6)	13 (9.8)	13 (9.9)
Prediabetic	1 (0.7)	0 (0.0)	0 (0.0)	0 (0.0)	0 (0.0)
Rheumatoid arthritis	4 (3.0)	4 (3.3)	14 (11.6)	10 (7.5)	22 (16.8)
Sleep apnea	20 (14.9)	18 (15.0)	25 (20.7)	13 (9.8)	13 (9.9)
Thyroid disease	8 (6.0)	5 (4.2)	6 (5.0)	6 (4.5)	5 (3.8)
Type 1 diabetes	2 (1.5)	2 (1.7)	6 (5.0)	5 (3.8)	5 (3.8)
Type 2 diabetes	16 (11.9)	13 (10.8)	17 (14.0)	27 (20.3)	23 (17.6)
Hearing loss – corrected	9 (6.7)	8 (6.7)	6 (5.0)	15 (11.3)	15 (11.5)
Hearing loss – uncorrected	3 (2.2)	7 (5.8)	4 (3.3)	7 (5.3)	4 (3.1)
Hearing loss – unknown	0 (0.0)	0 (0.0)	0 (0.0)	1 (0.8)	0 (0.0)
Other cardiovascular disease or heart condition	6 (4.5)	6 (5.0)	9 (7.4)	26 (19.5)	23 (17.6)
I have not been diagnosed with any of these health conditions	34 (25.4)	38 (31.7)	23 (19.0)	20 (15.0)	12 (9.2)

Abbreviations: COPD, chronic obstructive pulmonary disease; PLWAD, people living with Alzheimer's disease; SD, standard deviation.

**TABLE 2:** Quantitative survey: care partner characteristics (*N* = 265).

	Care partner respondents	
Question	Group 4 (*n* = 133)	Group 5 (*n* = 132)
**Age**		
*n*	133	132
Mean (SD)	54.1 (15.1)	55.0 (16.8)
Min‐max	21.0 to 93.0	22.0 to 90.0
**Sex assigned at birth**		
Female	37 (27.8)	54 (40.9)
Male	96 (72.2)	78 (59.1)
Intersex	0 (0.0)	0 (0.0)
Prefer not to answer	0 (0.0)	0 (0.0)
**Race or ethnicity (Please select all that apply)**		
African American or Black	45 (33.8)	43 (32.6)
Alaska Native, American Indian, or Native American	3 (2.3)	2 (1.5)
Asian or Asian American	4 (3.0)	9 (6.8)
Hispanic, Latina/o, Latine, or Latinx	21 (15.8)	25 (18.9)
Middle Eastern and/or North African	0 (0.0)	1 (0.8)
Native Hawaiian or Pacific Islander	0 (0.0)	0 (0.0)
White	64 (48.1)	54 (40.9)
A race or ethnicity not listed	1 (0.8)	0 (0.0)
Prefer not to answer	1 (0.8)	0 (0.0)
**Highest grade or level of education**		
Less than high school	0 (0.0)	1 (0.8)
High school diploma or equivalent (e.g., GED)	16 (12.0)	21 (15.9)
Associate's degree/technical school	16 (12.0)	11 (8.3)
Some college	24 (18.0)	28 (21.2)
College degree (e.g., BA, BS)	51 (38.3)	53 (40.2)
Some graduate school but no degree	6 (4.5)	3 (2.3)
Graduate or professional degree (e.g., MS, MD, PhD, JD)	20 (15.0)	15 (11.4)
Prefer not to answer	0 (0.0)	0 (0.0)
**What is your relationship to the PLWAD?**		
Spouse	35 (26.3)	31 (23.7)
Child of PLWAD	40 (30.1)	49 (37.4)
Another family member	36 (27.1)	28 (21.4)
Friend	12 (9.0)	13 (9.9)
Paid professional caregiver	10 (7.5)	10 (7.6)

Abbreviations: PLWAD, people living with Alzheimer's disease; SD, standard deviation.

Acknowledging that the characteristics for individuals with moderate or severe AD were reported by care partner respondents in Groups 4 and 5, the mean age of individuals at risk of or living with AD increased with increasing disease severity from 58.5 (Group 1) to 76.6 (Group 5) years, and the majority were female (52.1% to 59.0%). For care partners, the mean age was 54.1 to 55.0 years, most were male (59.1% to 72.2%), and most were the child (30.1% to 37.4%), spouse (23.7% to 26.3%), or other family member (21.4% to 27.1%) of the PLWAD. Most PLWAD in Groups 1 to 3 were living with spouses/partners (41.3% to 55.2%) or alone in their own home (35.1% to 42.1%), while PLWAD in Group 4 or 5 (i.e., with moderate or severe AD dementia) were most frequently living with spouses/partners in their own home (27.1% to 28.8%) or living with an adult child or children (24.1% to 28.0%). The highest education level most often reported for PLWAD in Groups 1, 2, and 3 was a college degree (26.1% to 30.8%), and for individuals with dementia in Groups 4 and 5, it was a high school diploma or equivalent (36.4% to 39.1%). Most PLWAD in Groups 1, 2, and 3 self‐reported concern that they had a problem with memory or thinking (81.3% to 96.7%) and had already discussed their memory or thinking problems with their healthcare providers (79.1% to 95.0%), who were most often primary care/general practitioners (53.3% to 61.9%) or neurologists (23.1% to 44.2%). The three most common health conditions across Groups 1 to 5 were high blood pressure (32.1% to 49.6%), depression (20.6% to 29.2%), and anxiety (18.0% to 32.1%).

### 3.2 Qualitative interviews: WMM concepts and conceptual model of disease (Stage 1)

All participants (*N* = 64), regardless of disease severity, reported on the highly symptomatic and consequential nature of living with AD. The majority (63% of PLWAD and 75% of care partners) indicated the descriptor “impact” (over “bother” or “interference”) offered the most information regarding how concepts may be most accurately evaluated, considering their daily life (Tables S6 and S7).

**FIGURE 1 alz71554-fig-0001:**
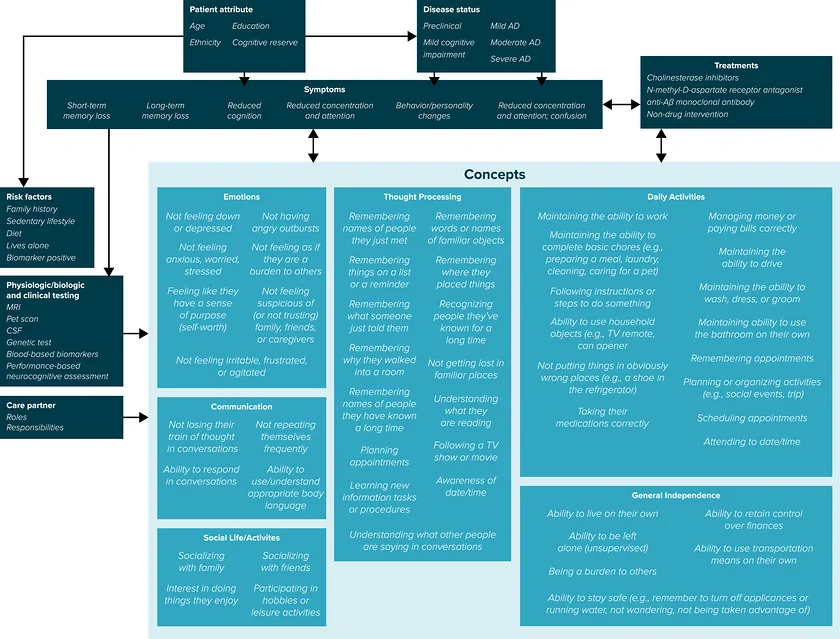
WMM conceptual model of disease. AD, Alzheimer's disease; CSF, cerebrospinal fluid; MRI, magnetic resonance imaging; PET, positron emission tomography; WMM, What Matters Most.

Participants endorsed all initial 42 WMM concepts as important. The conceptual model – with hypothesized domains of Thought Processing, Daily Activity, Emotion, General Independence, Communication, and Social Life/Activity – was consistently described as comprehensive and largely appropriate as originally presented. Some modifications were suggested (Figure S1, red shading), including four new candidate concepts, repositioning of existing concepts to describe the lived experience of AD better and recategorizing or separating multidimensional concepts to reflect participant interpretation of the domain or item more accurately (e.g., splitting “knowing the date and time” into “awareness of date/time” and “attending to date/time”). These interview findings informed the conceptual model, including an increase from 42 to 50 concepts across six domains (Figure 1).

While all WMM concepts were considered important, all participants were able to identify priorities among these concepts within and between WMM domains clearly. Participants, including those with MCI and mild AD dementia, performed a simple, computerized ranking assessment and provided clear rationale for their selections. Respondents also articulated clear preferences for a single most important WMM domain for hypothetical treatment benefits, with the thought processing domain identified most frequently (55%) in the combined respondent population (Figure S2). Notably, preferences differed between PLWAD and care partner respondents; for instance, only 46% of PLWAD indicated that thought processing was the most important domain compared with 62% of care partners, and communication was indicated as most important by 18% of PLWAD compared with 2% of care partners.

### 3.3 Quantitative survey pretesting (Stage 2a)

Following completion of the web‐based draft survey, all participants (*N* = 17) reported in cognitive debriefing interviews that the survey was easy to complete, and none reported malfunctions or difficulty navigating to the next screen or back to previous questions (Table S8). Most participants reported using a desktop or laptop computer (47.1%) or smartphone (41.2%) to complete the survey. All participants thought the length of the survey was acceptable, though a few noted that it should not be any longer.

Participants stated the survey questions were easy to follow, understand, and answer and that they were relevant to their lived experiences. All participants reported being able to answer the BWS prioritization questions, though a few participants reported that it was sometimes challenging because two or more of the concepts on the screen felt equally important to them or that some of the questions in the prioritization exercise felt repetitive. Some of the care partners reported challenges with understanding or remembering from one section to the next whether they were to indicate the amount of importance a concept had for the PLWAD for whom they provide care or for themselves. Using this feedback, instructions for the care partner survey were modified to clarify the perspective from which care partners should answer questions (e.g., “[P]lease click on the ONE concept that is MOST important TO YOU for the person you provide care for to either retain or be treated for”) and included on each screen.

### 3.4 WMM quantitative survey and priority ranking (Stage 2b)

In addition to an analysis of the full quantitative survey sample, the large sample size (*N* = 640) allowed comparisons between PLWAD and care partners, across AD groups, and among certain racial and ethnic groups. However, prioritization findings must be interpreted with caution, as (1) relative importance weights cannot be compared across domains and (2) the variability of the priority weights was not estimated, so the results do not support conclusions about statistical significance of differences.

#### 3.4.1 Full sample

**FIGURE 2 alz71554-fig-0002:**
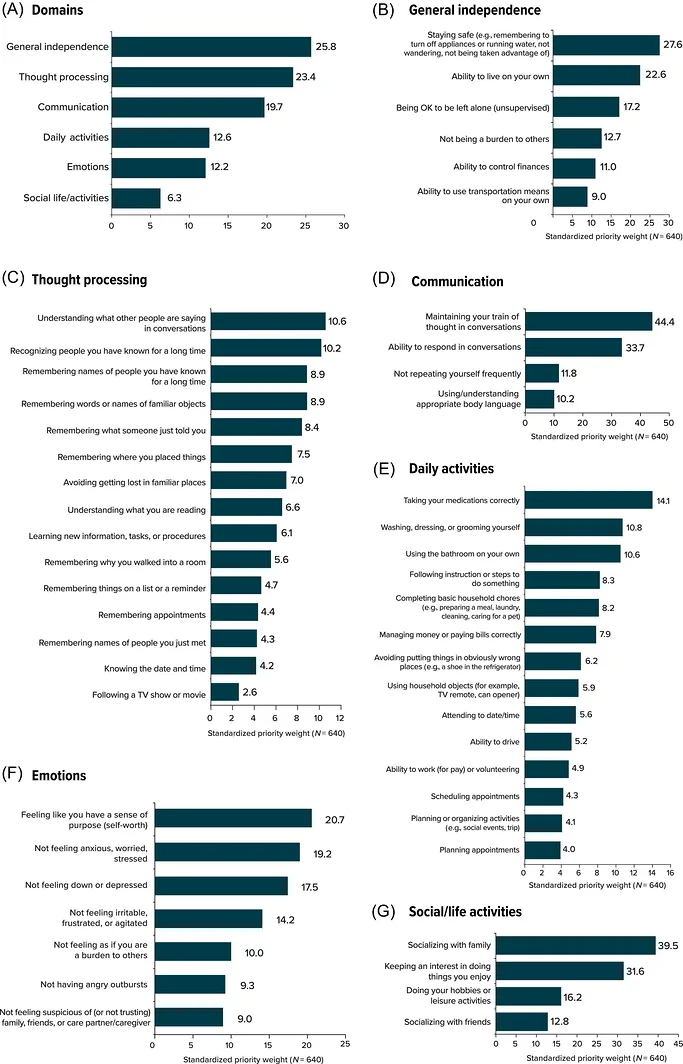
WMM priority ranking: Full sample (*N* = 640). *Note*: Standardized, relative priority weights sum to 100% for each set of WMM concepts or domains presented. Greater weights correspond to higher assigned priority, and the order of weight magnitudes indicates the ranking of concepts or domains, indicating the items of most and least priority. Panel A previously presented at the AAIC 2025 meeting, July 27–31, 2025, Toronto, Canada.^40^ AAIC, Alzheimer's Association International Conference; WMM, What Matters Most.

Priority ranking findings demonstrate PLWAD and care partners can and do prioritize among the WMM concepts and domains (Figure 2). Overall, for domains, respondents placed the highest priority on General Independence, followed by Thought Processing, Communication, Daily Activities, Emotions, and Social Life/Activities (Figure 2A).

Within‐domain concept ranking findings are presented in Figures 2B–G. Within each domain, respondents placed the highest priority on the concepts of “staying safe (for example, remembering to turn off appliances or running water, not wandering, not being taken advantage of)” (General Independence), “understanding what other people are saying in conversation,” followed closely by “recognizing people you have known for a long time” (Thought Processing), “maintaining your train of thought in conversation” (Communication), “taking your medications correctly” (Daily Activities), “feeling like you have a sense of purpose (self‐worth)” (Emotions), and “socializing with family” (Social Life/Activities).

#### 3.4.2 PLWAD and care partners

**FIGURE 3 alz71554-fig-0003:**
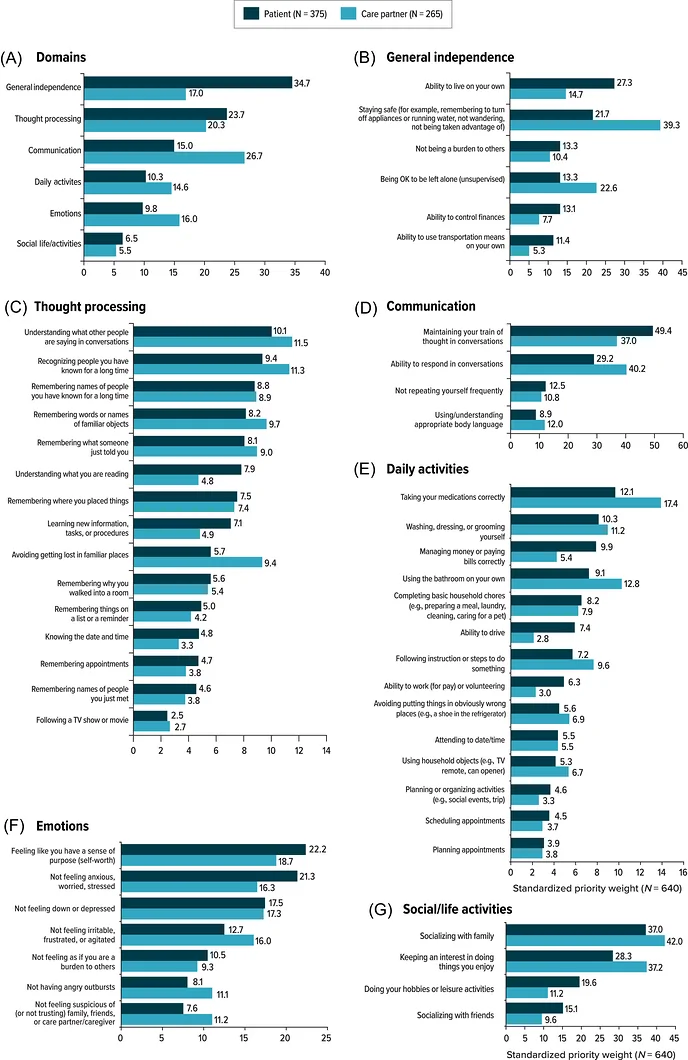
WMM priority ranking: PLWAD (*N* = 375) and care partners (*N* = 265). *Note*: Standardized, relative priority weights sum to 100% for each set of WMM concepts or domains presented. Greater weights correspond to higher assigned priority, and the order of weight magnitudes indicates the ranking of concepts or domains, indicating the items of most and least priority. Panel A previously presented at the AAIC 2025 meeting, July 27–31, 2025, Toronto, Canada.^40^ AAIC, Alzheimer's Association International Conference; PLWAD, people living with Alzheimer's disease; WMM, What Matters Most.

There were differences in prioritization observed between the PLWAD and care partner groups (Figure 3). However, with the differences in disease severity between PLWAD Groups 1 to 3 and care partner Groups 4 and 5 inherent in the study design, whether these differences are driven by perspective or disease severity cannot be discerned from these observations alone. Among domains, PLWAD selected General Independence as the highest priority, whereas care partners selected Communication; care partners also placed higher priority on the domains of Daily Activities and Emotions than did PLWAD (Figure 3A). Both similarities and differences were observed for within‐domain concept ranking by PLWAD and care partners (Figures 3B–G). Priorities differed notably in the General Independence and Communication domains. In the former, PLWAD prioritized “ability to live on your own” twice as much as care partners; conversely, “staying safe” was prioritized half as much by PLWAD as by care partners. In the Communication domain, “maintaining train of thought in conversation” was prioritized highest by PLWAD, followed by “ability to respond in conversations,” and the converse was observed for care partners.

Generally, PLWAD and care partners had similar prioritizations of the Thought Processing concepts, with the two highest‐priority items for both being “understanding what other people are saying in conversations” and “recognizing people you have known for a long time,” though care partners placed higher relative priority than PLWAD on both of these concepts. Among Daily Activities concepts, both PLWAD and care partners placed the highest priority on “taking your medications correctly,” though care partners’ relative priority weights were again greater than those for PLWAD. For the Emotions domain, PLWAD and care partners consistently prioritized “feeling like you have a sense of purpose (self‐worth),” followed by “not feeling anxious, worried, stressed” (second most important for PLWADs, third most important for care partners) and “not feeling down or depressed” (third most important for PLWADs, second most important for care partners). PLWAD and care partners ranked the Social Life/Activities concepts the same, placing the highest priority on “socializing with family,” followed by “keeping an interest in doing things you enjoy.”

#### 3.4.3 AD severity

**FIGURE 4 alz71554-fig-0004:**
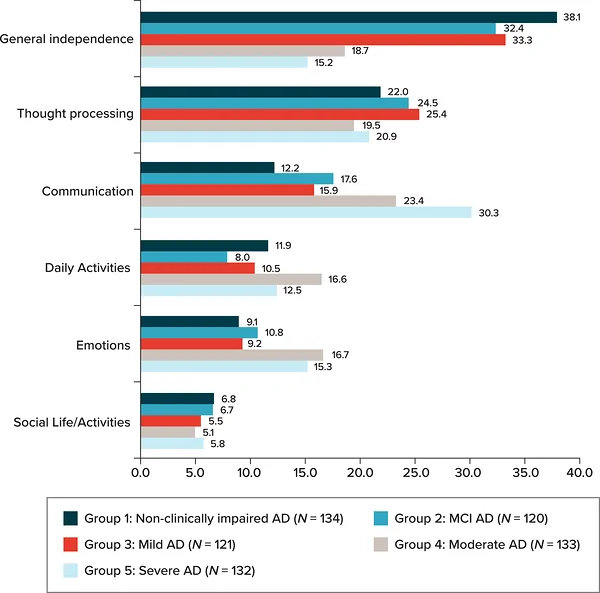
WMM priority ranking: AD severity subgroups. *Note*: Standardized, relative priority weights sum to 100% for each set of WMM concepts or domains presented. Greater weights correspond to higher assigned priority, and the order of weight magnitudes indicates the ranking of domains, indicating the domains of most and least priority. Within‐domain concept ranking presented in Figure S3. Previously presented at AAIC 2025 meeting, July 27–31, 2025, Toronto, Canada.^40^ AAIC, Alzheimer's Association International Conference; AD, Alzheimer's disease; MCI, mild cognitive impairment; WMM, What Matters Most.

Prioritization of domains differed for groups across the disease continuum (Figure 4). Priority weights for the General Independence domain decreased as AD severity increased, while weights for the Communication and Emotions domains tended to increase as severity increased. The Social Life/Activities domain was weighted similarly across all groups and was the lowest priority. Acknowledging sample size limitations for severity subgroups, concept prioritization trends within all domains differed with increasing disease severity (Figure S3).

#### 3.4.4 Race and ethnicity subgroups

**FIGURE 5:**
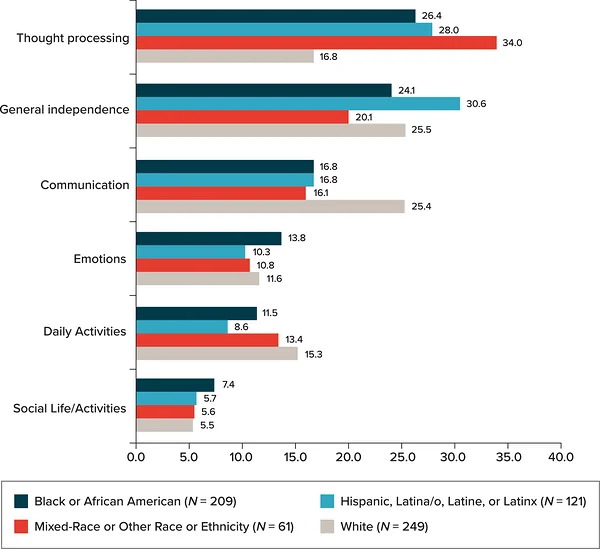
WMM priority ranking: race and ethnicity subgroups. *Note*: Standardized, relative priority weights sum to 100% for each set of WMM concepts or domains presented. Greater weights correspond to higher assigned priority, and the order of weight magnitudes indicates the ranking of domains, indicating the domains of most and least priority. Within‐domain concept ranking presented in Figure S4. Subgroups were defined by the respondents’ self‐reported racial and ethnic identity (for care partners, their self‐reported race/ethnicity was used, not that for the PLWAD for whom they care) and consisted of respondents identifying solely as Black or African American (*n* = 209); Hispanic, Latina/o, Latine, or Latinx (*n* = 121); mixed race or another race or ethnicity, including Alaska Native, American Indian, Native American, Asian, Asian American, Middle Eastern, North African, Native Hawaiian, Pacific Islander, or a race or ethnicity not listed in the survey (*n* = 61); or White (*n* = 249). Previously presented at AAIC 2025 meeting, July 27–31, 2025, Toronto, Canada.^40^ AAIC, Alzheimer's Association International Conference; PLWAD, people living with Alzheimer's disease; WMM, What Matters Most.

PLWAD and care partner groups were combined to increase subgroup size for analysis by race and ethnicity. Differences and similarities[Fig alz71554-fig-0001], [Fig alz71554-fig-0002], [Fig alz71554-fig-0003], [Fig alz71554-fig-0004] in domain prioritization were observed by race and ethnicity subgroups (Figure 5). For instance, the Thought Processing domain had the greatest priority weight for multiracial participants and was approximately two times that for White participants. The priority of the General Independence domain was greater for Hispanic and White participants than for Black and multiracial participants. Similarly, concept prioritization similarities and differences were observed within domains (Figure S4).

## 4 DISCUSSION

### 4.1 Overall findings

As part of the WMM research program, this mixed‐methods study provides important insights into the lived experience of AD and quantifies the priorities of individuals at risk for or with AD and care partners of people with more advanced AD, comprising a large, demographically diverse, and inclusive cohort representing the full AD severity spectrum.

Overall, PLWAD and care partners reported the lived experience of AD as both highly symptomatic and consequential and endorsed all WMM concepts as important to the lived experience. Qualitative findings informed refinement of the WMM conceptual model of disease, expanding from the original 42 to 50 concepts mapped across the original six hypothesized domains: General Independence, Thought Processing, Communication, Daily Activities, Emotions, and Social Life/Activities. These domains and the concepts within each one were prioritized using BWS in a quantitative survey, with the sample size of 640 PLWAD and care partners of people with more advanced disease, allowing comparison of prioritization as severity of symptoms progresses and among some racial and ethnic groups. The priority ranking findings demonstrate that individuals at risk for AD, with MCI, and with mild AD and care partners of people with more severe disease prioritized and meaningfully differentiated among specific WMM domains and concepts.

There were important similarities and differences in WMM domain and concept prioritization by PLWAD and care partners and by individuals of differing AD severity. Among PLWAD (Groups 1 to 3), the General Independence domain was consistently ranked the highest, followed by Thought Processing and Communication. However, prioritization of the other domains (Daily Activities, Emotions, Social Life/Activities) changed with increasing disease severity, and care partners of people with moderate and severe disease placed increasing importance on the Communication domain. Differences between PLWAD and care partners may reflect both the progression of disease and differences in observations by care partners in comparison with the PLWAD lived experience. Acknowledging that race and ethnicity subgroups were a combination of PLWAD and care partners, participants who identified as Black or African American and those who identified as Mixed Race or Other Race placed the greatest priority on the Thought Processing domain, whereas participants who identified as White or Hispanic or Latino placed the greatest priority on General Independence. These similarities are important to inform the relevance of WMM concept measurement, especially in the context of clinical trials, where maximizing the relevance of endpoints (“measuring What Matters Most”) is key for all decision‐makers. Further, these differences are important to inform future work examining underlying contributing factors and evaluating clinical care and the execution of clinical research protocols and schedules of assessments (i.e., targeted measurements and targeted interventions).

This work provides new insights for the field, and this study has numerous strengths. Especially noteworthy is the novel nature of the dataset. The study team is unaware of any other such study that has captured outcomes relevant to multiple decision‐makers from a large, diverse cohort reflective of the real‐world population of individuals affected by AD and likely to be recipients of new treatments in the clinic. For PLWAD and care partner participants, disease status was clinically confirmed, and a well‐developed, pretested survey was used to ensure high‐quality data collection.

The sampling strategy was designed to ensure representation of the full AD severity spectrum, as well as overall and within‐AD group representation of individuals across racial and ethnic backgrounds consistent with the US population.^1^ During survey recruitment, recruitment partners reported that Black and Hispanic participants were disproportionately excluded from the study due to a comorbid diagnosis of vascular dementia. A targeted review of the literature and expert consensus – including a non‐binding opinion from the FDA – supported expansion of eligibility criteria to allow a diagnosis of vascular dementia, which resulted in a more robust recruitment reflective of the US population. Nonetheless, limitations must be considered. As this study used a cross‐sectional sample and predefined severity groups, the findings may not be representative of the time between disease progression. Limitations inherent in observational and survey‐based studies (e.g., recall bias, misinterpretation, confounding variables) must be considered. For example, there may be potential confounding by reporter type (PLWAD vs care partners) or respondent bias, as only care partners completed surveys for Groups 4 and 5 due to the nature of disease severity. For PLWAD in Groups 1 to 3 in particular, there may also be a risk of confounding due to individual variability in the self‐awareness of their cognitive condition (e.g., anosognosia) that should be considered.^28^ Further, the topics discussed in these surveys may be sensitive to respondents, which may introduce difficulty or unwillingness to provide complete and accurate responses. The highest level of education of the PLWAD survey participants (college degree for Groups 1 to 3 [26.1% to 30.8%]; high school diploma or equivalent for Groups 4 and 5 [36.4% to 39.1%]) may be lower than typically observed in clinical studies, which could reflect differences in recruitment methods and/or in the characteristics of individuals willing to participate in different study types.^29^ Despite the overall representativeness of racial and ethnic identities and disease severity groups, subgroup sample sizes posed limitations on intergroup comparisons, especially at the within‐domain concept ranking level. Future directions include assessing whether systematically different priorities exist between or within subgroups and will be informed by the recruitment challenges and adaptations applied in the administration of the quantitative survey. These comparisons would provide increased understanding of how priorities may be affected by clinical and cultural differences.

### 4.2 Context

Insights into the priorities of PLWAD and care partners provide an important framework in which to consider meaningful benefits regarding symptoms, functioning, and outcomes. While emerging therapies for AD aim to demonstrate meaningful benefits by reaching various clinical thresholds, “meaningful” is often used without reference to a unit of measure specific to an outcome or inherently meaningful to PLWAD or care partners, and long‐established COAs are often anchored in clinician impressions, rather than those of PLWAD.^2^, ^3^, ^30^, ^31^, ^32^ The current study aligns with the approaches required to assess meaningful clinical benefit from a US regulatory perspective set forth by Buracchio et al.,^6^ including the emphasis on obtaining input from PLWAD and care partners as critical to understanding the perspective of clinical benefit.

The present findings describing WMM concepts of importance to PLWAD and care partners largely align with other studies similarly aiming to identify the most prevalent and burdensome symptoms, such as a recent large‐scale survey of individuals living with MCI or dementia, including AD (or respective care partners).^33^ However, this study had notable limitations (e.g., survey sample predominantly completed by care partners [76%]; respondents living with disease predominantly had MCI [72%]; predominantly White participants [94%]).^33^ Limitations such as these were considered in the study design across the WMM research program, and as a result, the recruitment and participation of PLWAD and care partners were evenly distributed across the disease severity spectrum and representative of diverse racial and ethnic identities within each disease severity group.

### 4.3 Future directions and conclusions

The quantitative survey assessed the impact of WMM concepts on lived experience and included participant completion of COAs to facilitate HRQOL and health economic assessments (EQ‐5D‐5L^34^, ^35^; Quality of Life in AD^36^, ^37^) and psychometric evaluation of the proposed domain structure (Cognitive Function Index [CFI] ^38^); these findings are not reported presently and are intended as the subject of future manuscripts. Evaluation of the concepts and their impacts can inform clinical care as well as selection of appropriate measurement tools for observational and clinical research across the continuum of disease. The ongoing WMM research program aims to use the insights from the quantitative survey to evaluate the relationship of WMM concepts to existing COAs. Insights from measuring the impact of concepts on daily life facilitate comparison and mapping of the concepts to COAs commonly used in clinical trials, and the inclusion of standardized COAs in the survey provides details on the relationship of WMM concepts to established measurements. Coupled with details on how individual priorities were established among concepts, WMM concepts could ultimately serve as the basis for a roadmap to identify existing COAs – as well as measurement gaps for novel COA development – for use in future AD prevention and treatment research and clinical trials that can capture treatment‐related meaningful changes.

Results and insights from the ongoing WMM research program have the potential to bridge the perspectives of PLWAD and care partners on “what matters most” with those of the AD community and key decision‐makers, including clinicians, payers, policymakers, and others touched by this disease. Findings from this phase of research provide critical insights into the lived experience of AD and the identification of meaningful treatment goals, which can ultimately inform the selection – or development – of relevant patient‐centric outcome measures and study endpoints aligning with best practices for assessing meaningful clinical benefit as AD treatments continue to emerge.

## CONFLICT OF INTEREST STATEMENT

C(D)R, EB‐W, DD, WLH, CB, GJD, DG, TE, LM, and CP are full‐time employees of RTI Health Solutions, which was retained by UsAgainstAlzheimer's to conduct the research that is the subject of this manuscript. Their compensation is unconnected to the studies on which they work. WLH is affiliated to research with the Karolinska Institute but receives no compensation from this affiliation. AH is an employee of Eli Lilly & Co. and may hold shares and/or stock options in the company. JT and DM report no conflicts of interest. LFC is an employee of the University of North Carolina at Chapel Hill. IK is a consultant serving as executive director of the LEAD Coalition and does not invest directly in companies working in the field, though he may hold mutual funds that include such companies. DL is an employee of Lappin Kramer LLC. TF and SS are employees of Faegre Drinker Consulting. JL and ES are employees of AbbVie, Inc., and may hold shares and/or stock options in the company. DS is an employee of Genentech and may hold shares and/or stock options in the company. RP was an employee of UsAgainstAlzheimer's at the time the research was conducted. Author disclosures are available in the Supporting Information.

## ETHICAL APPROVAL

This study was reviewed by the RTI International IRB and determined to meet the criteria for exemption from IRB review. It was performed in accordance with the ethical standards laid down in the 1964 Declaration of Helsinki and its later amendments. Informed consent was obtained from all participants in this study, and participants were compensated for their time.

## DISCLOSURE

Portions of the methods and data reported in this manuscript have been the subject of prior presentations at the Alzheimer's Association International Conference (AAIC) meetings in 2023 (July 16–20, Amsterdam, Netherlands^13^), 2024 (July 28–August1, Philadelphia, PA, USA^39^), and 2025 (July 27–31, Toronto, Canada^40^) and Clinical Trials on Alzheimer's Disease (CTAD) 2023 meeting (October24–27, Boston, MA, USA^41^).

## Supporting information



Supporting information

Supporting information

## Data Availability

Data from the qualitative study are primarily in the form of transcripts and will only be made available to the AD PACE Data Commons with random identifiers in order to protect participant privacy in accordance with the principles of the Belmont Report. Data from the quantitative research will also be stored in the AD PACE Data Commons associated with random identifiers.
