# Supplementary material for: Priorities of people living with Alzheimer's and care partners: What Matters Most?
Source: Alzheimers Dement. 2026 Aug 27;22(8):e71554. doi: 10.1002/alz.71554 (PMC13520114; doi:10.1002/alz.71554)
Supplement: Supplementary file 2 [file ALZ-22-e71554-s001.docx]

Priorities of people living with Alzheimer’s and care partners: What Matters Most?

Carla (DeMuro) Romano,^1^ Emily Bratlee-Whitaker,^1^ Ann Hartry,^2^ Jim Taylor,^3^ Leigh F. Callahan,^4^ Doreen Monks,^5^ Ian Kremer,^6^ Debra Lappin,^7^ Terry Frangiosa,^8^ Sanjyot Sangodkar,^8^ Jae Lee,^9^ Elaheh Shirneshan,^9^ Diana Slowiejko,^10^ Dana DiBenedetti,^1^ William L. Herring,^1,11^ Cooper Bussberg,^1^ Gabrielle J. Dardis,^1^ Diana Goss,^1^ Teresa Edwards,^1^ Lori McLeod,^1^ Christine Poulos,^1^ Russ Paulsen^12^

^1^RTI Health Solutions, Durham, NC, United States;

^2^Eli Lilly & Co, Indianapolis, IN, United States;

^3^Memory Advocate Peers (MAP), New York, NY, United States;

^4^Department of Medicine, University of North Carolina, Chapel Hill, NC, United States;

^5^Advocate and person living with Alzheimer’s disease, Livingston, NJ, United States; ^6^LEAD Coalition (Leaders Engaged on Alzheimer’s Disease), Washington, DC, United States;

^7^Lappin Kramer LLC, Denver, CO, United States;

^8^Faegre Drinker Consulting, Washington, DC, United States;

^9^AbbVie Inc., Chicago, IL, United States;

^10^Genentech, San Francisco, CA, United States

^11^Karolinska Institute, Stockholm, Sweden

^12^UsAgainstAlzheimer’s, Washington, DC, United States;

Corresponding Author:

Russ Paulsen, MA

[rpaulsen@usagainstalzheimers.org](mailto:rpaulsen@usagainstalzheimers.org)

UsAgainstAlzheimer’s

5614 Connecticut Ave NW, #288

Washington, DC 20015

United States

Running Head: What Matters in Alzheimer’s Disease

# SUPPLEMENTARY MATERIAL

Table S-1. AD Groups

| AD Group | | Clinical confirmation criteria | Approximate stage per  US FDA draft guidance^a^ |
| --- | --- | --- | --- |
| **Group 1:**  **At risk/preclinical** | Individuals with unimpaired cognition per self-report with evidence of AD pathology | Evidence of AD pathology determined by positive findings of amyloid positron emission tomography scan or cerebrospinal fluid lumbar puncture within the past 6 months | Similar to Stage 1 [1] |
| **Group 2:**  **MCI** | Individuals with mild cognitive impairment due to AD | Evidence of AD pathology as described above within the past 6 months and complaints of memory problems, losing or misplacing things, forgetting events or appointments, word-finding difficulties, etc. (based on chart notes or patient self-report) | Similar to Stage 2, Stage 3, and early Stage 4 [1] |
| **Group 3:**  **Mild AD** | Individuals with mild AD dementia | Evidence of mild AD as determined by a Mini-Mental State Examination (MMSE) score of approximately 20 to 24 [2], a physician’s assessment, or a comparable neuropsychological assessment within in the past 6 months | Similar to Stages 3-4 [1] |
| **Group 4:**  **Moderate AD** | Care partners of individuals with moderate AD dementia | Evidence of moderate AD as determined by an MMSE score of approximately 13 to 20 [2], a physician’s assessment, or a comparable neuropsychological assessment that can be used for AD staging within in the past 6 months | Stage 5 [1] |
| **Group 5:**  **Severe AD** | Care partners of individuals with severe AD dementia | Evidence of severe AD as determined by an MMSE score of 12 or less [2], a physician’s assessment, or comparable neuropsychological assessment within in the past 6 months | Stage 6 [1] |

AD = Alzheimer’s disease; FDA = Food and Drug Administration; MCI = mild cognitive impairment; PLWAD = people living with Alzheimer’s disease; US = United States.

^a^ US FDA draft guidance includes Stage 1 (“Patients with characteristic pathophysiological changes of AD but no evidence of clinical impact”), Stage 2 (“Patients with characteristic pathophysiological changes of AD and subtle detectable abnormalities on sensitive neuropsychological measures or subjective complaints of mild cognitive symptoms but no functional impairment”), Stage 3 (“Patients with characteristic pathophysiological changes of AD, generally more apparent detectable abnormalities on sensitive neuropsychological measures, and mild but detectable functional impairment”), and Stages 4, 5, and 6 (“Patients with overt dementia, progressing through mild, moderate, and severe stages”) [1].

Table S-2. Study Eligibility Criteria

| Study stage | Inclusion criteria | Exclusion criteria |
| --- | --- | --- |
| All stages | - Aged at least 18 years - Have a clinical diagnosis of AD (or identification of “at risk” for asymptomatic patients); qualitative survey: may include a diagnosis of vascular dementia^a^ - Provides self-report for classification into 1 of the 5 AD groups^b^ - Able to read, write, and understand English or Spanish | - PLWAD has a history of dementia (e.g., due to Parkinson’s disease, Huntington’s disease, FTD, Lewy body dementia, vascular dementia)^a^ - PLWAD has a recent (≤ 1 year) history of traumatic brain injury or cerebral vascular accident/stroke that is considered a primary cause of dementia |
| Qualitative and cognitive debriefing interviews | - Be able to participate in a 1-hour in-person (qualitative) or virtual (cognitive debriefing) interview | - PLWAD has any mental or other medical condition that the patient’s physician feels will interfere with the patient’s ability to engage in an interview |

AD = Alzheimer’s disease; FDA = Food and Drug Administration; FTD = frontotemporal dementia; PLWAD = people living with Alzheimer’s disease.

^a^ Quantitative survey: prior to 22 November 2024, participants with vascular dementia were excluded; subsequently, participants with vascular dementia were eligible for inclusion following FDA confirmation of expanded inclusion criteria.

^b^ Eligible qualitative interview participants included PLWAD in Groups 1-3 and care partners of PLWAD in Groups 1-5 included. Eligible quantitative survey participants included PLWAD in Groups 1-3 and care partners of PLWAD in Groups 4 and 5.

Table S-3. Recruitment Partner Descriptions

| Recruitment partner^a^ | Description |
| --- | --- |
| - UsAgainstAlzheimer’s (USA2): A-LIST Insight Series | UsA2’s A-LIST is an online, research-ready community of more than 8,000 people who self-identified with (or at risk for) Alzheimer’s disease, other dementias, or Mild Cognitive Impairment; current and former care partners; and people concerned about brain health. The A-LIST is conducting the ongoing What Matters Most Insights Survey Series study on issues that are most meaningful to those living with Alzheimer’s, other dementias and caregivers. |
| - Global Market Research Group (GMRG) - Sago | Qualitative research recruitment centers utilizing existing databases, preconsented panels for research, and referrals from partner clinicians. |
| - Paradigm Clinical Research - Tandem | Clinical trial sites utilizing prospective recruitment methods while screening potential participants |

^a^ Not all recruitment partners were involved in every stage or for the entire duration of the study.

Table S-4. Qualitative Interview Participant Characteristics (PLWAD, N = 24)

| Characteristic (PLWAD) | Group 1 (n = 11) | Group 2 (n = 7) | Group 3 (n = 6) | Group 4 (N/A) | Group 5 (N/A) | | | PLWAD overall (N = 24) |
| --- | --- | --- | --- | --- | --- | --- | --- | --- |
| Age (years) |  |  |  |  | |  |  | |
| Mean (SD) | 52.7 (5.7) | 74.1 (5.4) | 67.8 (10.6) | - | | - | 62.8 (11.8) | |
| Range | 47-66 | 64-81 | 52-80 | - | | - | 47-81 | |
| Sex assigned at birth, n (%) |  |  |  |  | |  |  | |
| Female | 10 | 5 | 3 | - | | - | 18 (75%) | |
| Male | 1 | 2 | 3 | - | | - | 6 (25%) | |
| Race and ethnicity, n (%) |  |  |  |  | |  |  | |
| African American or Black | 5 | 1 | 2 | - | | - | 8 (33%) | |
| Asian or Asian American | 0 | 1 | 0 | - | | - | 1 (4%) | |
| Hispanic, Latin American, Latine, or Latinx | 3 | 0 | 1 | - | | - | 4 (17%) | |
| White | 3 | 5 | 3 | - | | - | 11 (46%) | |
| Current employment status, n (%) |  |  |  |  | |  |  | |
| Employed full time | 3 | 0 | 1 | - | | - | 4 (17%) | |
| Employed part time | 7 | 0 | 0 | - | | - | 7 (29%) | |
| Retired | 1 | 7 | 4 | - | | - | 12 (50%) | |
| Disabled | 0 | 0 | 1 | - | | - | 1 (4%) | |
| Highest level of education, n (%) |  |  |  |  | |  |  | |
| High school diploma or equivalent (GED) | 1 | 1 | 2 | - | | - | 4 (17%) | |
| Associate’s degree/technical school | 1 | 0 | 0 | - | | - | 1 (4%) | |
| Some college | 6 | 2 | 2 | - | | - | 10 (42%) | |
| College degree | 3 | 4 | 1 | - | | - | 8 (33%) | |
| Graduate or professional degree | 0 | 0 | 1 | - | | - | 1 (4%) | |
| Total household income before tax and deductions in 2022, n (%) |  |  |  |  | |  |  | |
| $25,000 to $49,999 | 2 | 1 | 1 | - | | - | 4 (17%) | |
| $50,000 to $74,999 | 2 | 2 | 2 | - | | - | 6 (25%) | |
| $75,000 to $99,999 | 0 | 0 | 0 | - | | - | 0 (0%) | |
| $100,000 to $149,999 | 2 | 0 | 0 | - | | - | 2 (8%) | |
| $150,000 to $199,999 | 1 | 0 | 0 | - | | - | 1 (4%) | |
| $200,000 or more | 0 | 0 | 0 | - | | - | 0 (0%) | |
| Prefer not to answer | 4 | 4 | 3 | - | | - | 11 (46%) | |
| Current marital status, n (%) |  |  |  |  | |  |  | |
| Single | 1 | 1 | 1 | - | | - | 3 (13%) | |
| Married | 6 | 3 | 5 | - | | - | 14 (58%) | |
| Living with partner | 3 | 0 | 0 | - | | - | 3 (13%) | |
| Divorced or separated | 1 | 0 | 0 | - | | - | 1 (4%) | |
| Widowed/surviving partner | 0 | 3 | 0 | - | | - | 3 (13%) | |
| Current living arrangement, n (%) |  |  |  |  | |  |  | |
| Alone, in own home or apartment | 1 | 3 | 1 | - | | - | 5 (21%) | |
| With spouse/partner | 9 | 3 | 5 | - | | - | 17 (71%) | |
| With children | 1 | 1 | 0 | - | | - | 2 (8%) | |

AD = Alzheimer’s disease; PLWAD = people living with Alzheimer’s disease; SD = standard deviation.

Table S-5. Qualitative Interview Participant Characteristics (Care Partners, N = 40)

| Characteristic (Care partners) | | Group 1 (n = 5) | | Group 2 (n = 9) | | Group 3 (n = 10) | | Group 4 (n = 8) | | Group 5 (n = 8) | | Care partners overall (N = 40) |
| --- | --- | --- | --- | --- | --- | --- | --- | --- | --- | --- | --- | --- |
| Age (years) | | | | | | | | | | | | |
| Mean (SD) | 41.2 (12.6) | | 54.2 (17.3) | | 59.4 (16.5) | | 50.6 (4.3) | | 55.5 (8.2) | | 53.4 (13.7) | |
| Range | 20-53 | | 29-74 | | 24-79 | | 44-57 | | 41-68 | | 20-79 | |
| Sex assigned at birth, n (%) | | | | | | | | | | | | |
| Female | 5 | | 5 | | 6 | | 6 | | 6 | | 28 (70%) | |
| Male | 0 | | 4 | | 4 | | 2 | | 2 | | 12 (30%) | |
| Race and ethnicity, n (%) | | | | | | | | | | | | |
| African American or Black | 3 | | 2 | | 3 | | 2 | | 1 | | 11 (28%) | |
| Asian or Asian American | 0 | | 1 | | 0 | | 0 | | 1 | | 2 (5%) | |
| Hispanic, Latin American, Latine, or Latinx | 2 | | 0 | | 2 | | 3 | | 1 | | 8 (20%) | |
| White | 0 | | 6 | | 5 | | 3 | | 5 | | 19 (48%) | |
| Relationship to patient, n (%) | | | | | | | | | | | | |
| Parent | 0 | | 0 | | 1 | | 1 | | 1 | | 3 (8%) | |
| Another family member | 5 | | 9 | | 9 | | 7 | | 6 | | 36 (90%) | |
| Friend or another nonpaid professional caregiver | 0 | | 0 | | 0 | | 0 | | 1 | | 1 (3%) | |
| Current employment status, n (%) | | | | | | | | | | | | |
| Employed full time | 4 | | 2 | | 6 | | 7 | | 4 | | 23 (58%) | |
| Employed part time | 1 | | 2 | | 1 | | 0 | | 1 | | 5 (13%) | |
| Retired | 0 | | 3 | | 3 | | 0 | | 1 | | 7 (18%) | |
| Unemployed | 0 | | 2 | | 0 | | 1 | | 2 | | 5 (13%) | |
| Highest level of education, n (%) | | | | | | | | | | | | |
| High school diploma or equivalent (GED) | 0 | | 0 | | 1 | | 0 | | 1 | | 2 (5%) | |
| Associate’s degree/‌technical school | 1 | | 1 | | 2 | | 0 | | 1 | | 5 (13%) | |
| Some college | 1 | | 3 | | 3 | | 1 | | 2 | | 10 (25%) | |
| College degree | 3 | | 5 | | 3 | | 7 | | 4 | | 22 (55%) | |
| Graduate or professional degree | 0 | | 0 | | 1 | | 0 | | 0 | | 1 (3%) | |
| Total household income before tax and deductions in 2022, n (%) | | | | | | | | | | | | |
| $25,000 to $49,999 | 1 | | 2 | | 2 | | 0 | | 1 | | 6 (15%) | |
| $50,000 to $74,999 | 0 | | 2 | | 5 | | 2 | | 1 | | 10 (25%) | |
| $75,000 to $99,999 | 1 | | 0 | | 0 | | 3 | | 3 | | 7 (18%) | |
| $100,000 to $149,999 | 3 | | 1 | | 0 | | 1 | | 1 | | 6 (15%) | |
| $150,000 to $199,999 | 0 | | 1 | | 2 | | 1 | | 0 | | 4 (10%) | |
| $200,000 or more | 0 | | 0 | | 0 | | 0 | | 1 | | 1 (3%) | |

AD = Alzheimer’s disease; PLWAD = people living with Alzheimer’s disease; SD = standard deviation.

Table S-6. Qualitative Interviews: Contextualizing WMM Constructs (PLWAD, N = 24)

| Concept, n (%) | Group 1  (n = 11) | Group 2  (n = 7) | Group 3  (n = 6) | Group 4 (N/A) | Group 5 (N/A) | Total ^a^ (N = 24) |
| --- | --- | --- | --- | --- | --- | --- |
| Bother vs. interfere |  |  |  |  |  |  |
| Prefers bother | 2 | 2 | 2 | - | - | 6 |
| Prefers interfere | 5 | 3 | 3 | - | - | 11 |
| Prefers impact | 0 | 1 | 0 | - | - | 1 |
| Prefers concern | 1 | 0 | 1 | - | - | 2 |
| Endorsed neither bother nor interfere | 2 | 0 | 0 | - | - | 2 |
| Impact vs. interfere |  |  |  |  |  |  |
| Prefers impact vs. interfere | 9 | 2 | 4 | - | - | 15 |
| Prefers interfere vs. impact | 0 | 2 | 0 | - | - | 2 |
| Neutral on preferring impact vs. interfere | 0 | 0 | 1 | - | - | 1 |
| Endorsed neither impact nor interfere | 2 | 1 | 1 | - | - | 4 |

AD = Alzheimer’s disease; PLWAD = people living with Alzheimer’s disease; WMM = What Matters Most.

^a^ Two patients did not respond to bother vs. interfere question; two patients did not respond to impact vs. interfere question.

Table S-7. Qualitative Interviews: Contextualizing WMM Constructs (Care Partners, N = 40)

| Concept, n (%) | Group 1 (n = 5) | Group 2 (n = 9) | Group 3 (n = 10) | Group 4 (n = 8) | Group 5 (n = 8) | Total ^a^ (N = 40) |
| --- | --- | --- | --- | --- | --- | --- |
| **Bother vs. interfere** |  |  |  |  |  |  |
| Prefers bother | 0 | 2 | 1 | 2 | 2 | 7 |
| Prefers interfere | 1 | 2 | 4 | 3 | 3 | 13 |
| Prefers both bother and interfere | 0 | 1 | 0 | 0 | 1 | 2 |
| Prefers impact | 0 | 3 | 3 | 0 | 0 | 6 |
| Prefers concern | 3 | 1 | 0 | 1 |  | 5 |
| Endorsed neither bother nor interfere | 0 | 0 | 1 | 1 | 1 | 3 |
| **Impact vs. interfere** |  |  |  |  |  |  |
| Prefers impact | 4 | 7 | 8 | 6 | 5 | 30 |
| Prefers interfere | 0 | 0 | 1 | 0 | 1 | 2 |
| Neutral on preferring impact vs. interfere | 0 | 0 | 0 | 0 | 1 | 1 |
| Endorsed neither impact nor interfere | 1 | 0 | 0 | 1 | 0 | 2 |
| Prefers bother vs. impact or interfere | 0 | 1 | 0 | 0 | 0 | 1 |

AD = Alzheimer’s disease; WMM = What Matters Most.

^a^Four care partners did not respond to the bother vs. interfere question; four care partners did not respond to impact vs. interfere question.

Table S-8. Cognitive Interview Results

| Survey component | Response | Revision |
| --- | --- | --- |
| General feedback | Easy to understand and answer  Acceptable length  Relevant to experience as a PLWAD or care partner of a PLWAD | N/A: Maintain current appearance, formatting, content, and length |
| Introductory items | Respondents reported introduction was acceptable and informative and reported no concerns | No change |
| Symptoms and behaviors of AD | Clarify how care partners should answer questions about impacts of symptoms and behaviors associated with AD on the lives of the persons for which they provide care  Care partners may not always have insight into emotions and impact of emotions experienced by PLWAD  Add response options for care partners for items that are not applicable to PLWAD | Clarification statement and instruction added to Impact Questions for Groups 4-5  “Unsure” option added for items in Emotion domain for Groups 4-5  “Not applicable” options added to items “Ability to Drive,” “Managing money or paying bills correctly,” and “Ability to control finances” |
| Most and least important symptoms and behaviors of AD | Respondents were unsure and/or worried to use the “Exit Ranking” function | Remove “Exit Ranking” option |
| COA instruments |  |  |
| CFI | Concern expressed with remembering the timeframe respondents are to consider (1 year) | Include instruction “Answer all questions in comparison to 1 year ago” on each screen |
| EQ-5D-5L | Some reported difficulties with slider control | No change (required by license) |
| QOL-AD | Respondents could respond appropriately | No change |
| Time spent providing care | Concerns expressed for accuracy or unwillingness to estimate | Removed from survey |

AD = Alzheimer’s disease; CFI = cognitive function index; COA = clinical outcome assessment; N/A = not available; PLWAD = people living with Alzheimer’s disease; QOL-AD = Quality of Life in AD.

Figure S-1. Refined Conceptual Model of Disease

AD = Alzheimer’s disease. CSF = cerebrospinal fluid; CTAD = Clinical Trials on Alzheimer’s Disease. MRI = magnetic resonance imaging; PET = positron emission tomography.

Note: Red shading indicates new, refined, or repositioned concepts within the conceptual disease model. Previously presented at the CTAD 2023 meeting, 24-27 October 2023, Boston, MA, US [3].

Figure S-2. Most Meaningful Treatment Benefit Domain

CTAD = Clinical Trials on Alzheimer’s Disease; PLWAD = people living with Alzheimer’s disease.

Note: Previously presented at the CTAD 2023 meeting, 24-27 October 2023, Boston, MA, US [3].

Figure S-3. WMM Priority Ranking (Within-Domain): AD Severity Subgroups

AD = Alzheimer’s disease; MCI = mild cognitive impairment; WMM = What Matters Most.

Note: Standardized, relative priority weights sum to 100% for each set of WMM concepts or domains presented. Greater weights correspond to higher assigned priority and the order of weight magnitudes indicate the ranking of concepts or domains, indicating the items of most and least priority.

Figure S-4. WMM Priority Ranking (Within-Domain): Race and Ethnicity Subgroups

AD = Alzheimer’s disease; MCI = mild cognitive impairment; WMM = What Matters Most.

Note: Standardized, relative priority weights sum to 100% for each set of WMM concepts or domains presented. Greater weights correspond to higher assigned priority and the order of weight magnitudes indicate the ranking of concepts or domains, indicating the items of most and least priority.

SUPPLEMENTAL REFERENCES

1.	US Food and Drug Administration. Early Alzheimer’s disease: developing drugs for treatment: draft guidance. 2024. <https://www.fda.gov/regulatory-information/search-fda-guidance-documents/early-alzheimers-disease-developing-drugs-treatment>. Accessed 4 September 2025

2.	Alzheimer’s Association. Tests for Alzheimer’s disease and dementia. 2023. <https://www.alz.org/alzheimers-dementia/diagnosis>. Accessed June 12, 2023

3.	Romano C.D., Bratlee-Whitaker E., Herring W.L., Callahan L.F., Raimundo K., Taylor J., et al. Measuring what matters most to people living with Alzheimer’s disease and care partners: What Matters Most qualitative research. Poster presented at Clinical Trials on Alzheimer's disease (CTAD); 24-27 October 2023. Boston MA. 2023.
